# Surge in Economic Growth of Pakistan: A Case Study of China Pakistan Economic Corridor

**DOI:** 10.3389/fpsyg.2022.900926

**Published:** 2022-08-08

**Authors:** Maryam Farooq, Zia-ur-Rehman Rao, Muhammad Shoaib

**Affiliations:** ^1^Institute of Business and Management, University of Engineering and Technology, Lahore, Pakistan; ^2^Hailey College of Commerce, University of the Punjab, Lahore, Pakistan; ^3^Department of Computer Sciences, University of Engineering and Technology, Lahore, Pakistan

**Keywords:** IT infrastructure, FDI, human capital, transport infrastructure, economic growth

## Abstract

China Pakistan Economic Corridor (CPEC) is considered a massive investment that can change the economic scenario of Pakistan. The purpose of the study is to examine the contribution to the economic growth of the sectors where CPEC is investing. This research uses time-series data for 31 years to investigate the impact of macro-economic variables like foreign direct investment (FDI), human capital investment (HCI), transport investment, and information communication technology (ICT) on the economic growth of Pakistan. The results of Fully Modified Ordinary Least Square Regression Specification (FMOLS) show a positive nexus between FDI, HCI, and economic growth while economic growth and ICT show a negative relationship. The results for the impact of transportation infrastructure on economic growth are statistically insignificant. This research suggests that an increased focus on building knowledge, expertise, and skillset of human resources will help in reaping the benefits of CPEC’s investment. Future researchers can increase the period of the study to ascertain the implicit or explicit impact of CPEC on economic growth. The results also suggest that policymakers and researchers should focus on developing human capital to reap the investment benefits of CPEC.

## Introduction

The main idea of the economic corridor was initiated from the concept of the transport corridor. When these corridors extend to cover regions, benefits from increased investment and multilateral trade can be witnessed. However, a great deal of effort and infrastructure is required for retaining and improving such transportation networks. Therefore, a transport corridor mainly concentrating on upgraded infrastructure was established as an Economic Corridor (Arif, 2015). One Belt One Road (OBOR) is viewed as China’s strategic initiative to play power on the global level. As a part of China’s OBOR initiative, the China-Pakistan Economic Corridor (CPEC) is expected to provide financial and economic incentives to Pakistan. The importance of CPEC cannot be denied by both countries as Pakistan needs it to eradicate its economic, social, and energy problems, and it is needed by China because it requires safe and improved trade routes that can be used for oil supply from the Middle East countries to China (Ramay, 2016).

Amidst the crippling economic condition of Pakistan, CPEC is considered an investment that is expected to improve Pakistan’s economic growth. CPEC is China’s biggest foreign direct investment deal to invest in Pakistan (Shah, 2015). A huge amount is going to be spent on transportation, infrastructure, energy, and industrial zones. FDI is now commonly recognized to bring economic benefits along with technology, foreign exchange, competition, new market access, and capital (Crespo and Fontoura, 2007). This investment through CPEC makes up almost 20% of Pakistan’s Gross domestic product (GDP) (Stevens, 2015). In recent years, particularly from 2013 to 2014, Pakistan has faced a decline in FDI from the US in comparison with 2008; in such a demise, the economic corridor is expected to be an opportunity to increase economic growth (Board of Investment, Government of Pakistan, 2015). Full benefits of CPEC will be achieved by 2030 yet some rail, road, and energy projects are expected to be completed in the next few years (Ali and Faisal, 2017).

According to a report (Andrés et al., 2013) despite huge amounts being invested by the private sector, Pakistan’s infrastructure services are always insufficient to fulfill domestic needs. The report suggested increasing spending of GDP on telecommunication, electricity, and transport sectors by.71, 5.5, and 1.23%, respectively, until 2020. Pakistan’s economic growth and exports are suffering from a lack of necessary infrastructure. Recently, FDI increased up to 56% in Pakistan mainly due to increased investment in infrastructure through CPEC. The transport and electricity sectors have attracted huge amounts in form of FDI for the projects that are still under construction (Ahmed, 2017). Information and communication technology (ICT) infrastructure plays a substantial role in catalyzing economic growth, especially in today’s era of internet and mobile telecommunication (Lee et al., 2014; Rohman and Bohlin, 2014; Pradhan et al., 2016). Technology transfer from developed countries to developing countries is one of the mechanisms, by which FDI contributes to the economic growth of a country (Balasubramanyam et al., 1996). Likewise, Romer (1990) considered FDI as a medium of technological and economic growth. To benefit from the latest technology and ICTs, Van Reenen et al. (2010) recommended that developing countries like Pakistan should invest more in the training and education sector and CPEC is going to invest in this sector to provide Pakistan with the fifth route for its telecommunication traffic.

According to past studies, the researchers recommend investigating the impact of investment in transportation projects at the corridor level (Berechman et al., 2006). However, only increasing seaports and airports can result in traffic congestion on roads if roads are not properly constructed to cater to increased needs (Diaz et al., 2016), therefore, there is a need to investigate the impact of different modes of transportation infrastructure on economic growth. Economic Growth (EG) can be instigated by having a good transportation infrastructure, although it is not mandatory to find a positive impact on EG by all transportation modes (Ding, 2013; Diaz et al., 2016). Similarly, contrasting results between theoretical and empirical studies, showing a positive and negative nexus between FDI and economic growth are found (Ray, 2012; Akalpler and Adil, 2017). This investment is expected to improve the economic condition of Pakistan, as a researcher (Bucci, 2014) claimed that all types of capital investments, namely, human, physical, and innovational capital, lead to economic benefits.

Although CPEC is considered to play a vital role in the economic development of Pakistan, less attention is being paid to its sustainability and the spillover effect of these projects on human capital sustainability. CPEC is a project of massive investment, and such projects lead toward sustainable development (Zhuang et al., 2021) because infrastructural development affects all economic, social, human capital, and environmental policies and activities. Sustainability attempts to satisfy three major performance indicators, i.e., human, environment, and economic (Di Fabio and Peiroì, 2018). However, the human dimension of sustainability is often ignored (Pfeffer, 2010; Abid et al., 2020).

Since CPEC is a mega-investment project in Pakistan, it has the potential to change the economic scenario of not only the country but also the whole region. It is the need of the hour to investigate the sectors where investment and correct policy measures should be applied to cultivate the real economic benefits behind the CPEC. This research, therefore, is needed to identify which investment among human capital, information technology, transportation, and foreign direct investment be given prime importance. To recommend policies to make Pakistan a hub for foreign investment and to make it reap the benefits of infrastructure, information technology, enhanced human capital investments, and other economic benefits associated with CPEC; this research is much needed. To reap the benefits of this mega project, it needs time to analyze the impact of this investment on factors that will help Pakistan’s economy to benefit from it to its fullest potential. Therefore, this research is aimed at having a comprehensive study that can integrate economic changes occurring due to CPEC and provide relevant answers to the role of human capital investment, transport infrastructure, information communication technology, and foreign direct investment on economic growth.

This research attempts to estimate the impact of foreign direct investment, human capital, ICT, and transport infrastructure on Pakistan’s economic growth in connection with CPEC by taking data from FY-1985 to FY-2018 using fully modified ordinary least squared (FMOLS). Although CPEC regional integration analysis calls for including other countries in the analysis, the present investigation is limited to the approximation of the implicit impact of CPEC on Pakistan’s economy only. The results show a positive significant impact of FDI and human capital development on the economic growth of Pakistan. The benefits of all investments coming through CPEC can be realized if Pakistan develops and train its labor with the required knowledge and skills, whereas a negative relation between ICT and the economic growth of Pakistan is found. The study shows an insignificant relationship between the density of roads and economic growth during the selected time frame enhancing Pakistan’s concern to consider the route very carefully if it wants this project to provide expected benefits. This paper comprises six sections: introduction, literature review, methodology, discussions, conclusion and implications, and limitations and future recommendations.

## Literature Review

A transport corridor is considered a network within one country or between any two countries to connect economic centers (Khan et al., 2015). OBOR is viewed as China’s strategic initiative to play as power on a global level. This endeavor was started by China’s president XI Jinping in 2013 as a part of its economic integration of Eurasia through networks of roads (Aneja, 2016). OBOR is divided into two parts: the maritime Silk Road and Silk Road economic belt. As a part of China’s OBOR initiative (McCartney, 2021), CPEC is going to be extremely important for China and Pakistan in competition with territorial and global nations (Ali, 2016).

According to an announcement by a government official, the initial investment in the project was almost $46 billion, which increased up to $55 billion at first and later up to $62 billion (The Express Tribune, 2017). It would take almost $34 billion to invest in energy projects that are expected to contribute almost 17,000 MW of electricity, whereas the remaining $12 billion investment would be focused on infrastructure development out of the initial $46 (Shah, 2015). CPEC is composed of three types of projects based on the duration including short-term projects expected to be completed by 2017, medium-term projects that would be completed by 2025, and long-term projects that are expected to be completed by 2030 (Jawad, 2013).

The CPEC is China’s biggest foreign direct investment deal to invest in Pakistan (Shah, 2015). A huge amount is going to be spent on transportation, infrastructure, energy, and industrial zones (Abid and Ashfaq, 2015). The authors (Ali and Asghar, 2016) examined the sectoral impact of FDI on EG by taking the manufacturing, services, and agricultural sectors of Pakistan. Using the standard error model, they concluded that only two sectors, agricultural and manufacturing, have a significant positive impact on the outcome variables, stressing more to use the manufacturing sector as an option to enhance economic growth. Younus et al. (2014) used Two-Stage Least Squares estimation techniques and concluded a positive relation between FDI and the economic growth of Pakistan. Falki (2009) found a negative relation betwixt FDI inflows and the GDP of Pakistan from 1980 to 2006 based on endogenous growth theory. Atique et al. (2004) concluded that FDI plays a significant role in the economic growth of Pakistan particularly under the export regime between 1970 and 2001. The said phenomenon is explained by various studies; Nabi et al. (2022) investigated the impact of ICT, trade, and financial development on economic growth using ARDL, and Majeed et al. (2021) concluded that low- to middle-income countries involved in belt and road project tend to attract larger amounts of FDI, Bahrini and Qaffas (2019) compared the impact of ICT on economic growth between Middle Eastern and Sub Sharan African countries, Ibrahim and Alagidede (2018) concluded that a larger amount of finance, as well as a high level of per capital income, and human capital is needed for long-run economic growth. Latif et al. (2018) used OLS, FMOLS, and DOLS to examine the contribution of ICT, FDI, and globalization on economic growth, Niebel (2018) compared the impact of ICT on economic growth among developed, developing, and emerging economies. McCartney (2022) stated that owing to a lack of sound industrial policy CPEC may not have a revolutionary impact on Pakistan’s economy. McCartney (2021) stressed linking the economic outcome of CPEC with increased economic activity and prosperity in Western China. Javid (2019) used FMOLS to conclude that the impact of public infrastructure investment on Pakistan’s economy is better than the private sector’s investment. Khan and Liu (2019) investigated various challenges associated with CPEC including geopolitical environment, political instability, debt, terrorism, and issues with neighboring countries.

The authors (Padilla-Perez and Nogueira, 2016) stated that developing countries have witnessed an increasing trend of FDI in recent years. Both large and small economies have seen a trend of investing abroad by their domestic businesses. The researchers (Akalpler and Adil, 2017) concluded a negative relationship between GDP, gross savings, FDI, and international trade. A financial development index was developed for India in this research, and an investigation of FDI and EG showed a negative relationship both in a long and short period (Hye, 2011), while another study supports a positive relationship between FDI and EG in Pakistan (Chaudhry et al., 2013). In contrast, some studies found no link between FDI and EG, particularly in developing economies (Bende-Nabende et al., 2003); whereas a systemic link was investigated (Azman-Saini et al., 2010) between FDI and EG and concluded that there is no direct effect of FDI on EG.

Both developed and developing countries experienced a boost in their economic growth due to urbanization (Armeanu et al., 2021). Cities developed with investment in energy, transport, and infrastructure sectors attract a large amount of talent because of more economic opportunities. CPEC is expected to provide cooperation in infrastructure, telecommunication, energy, transportation sectors, and socio-economic development (Ahmed and Azam, 2016; Tasneem, 2018), some researchers (Ahmed et al., 2017) are focused on human resource development in the wake of CPEC. Pakistan neglected human resource development for decades (Abbassi and Burdey, 2008; Asrar-ul-Haq, 2015). The researchers (Ahmed et al., 2017) claimed that a proper human resource development policy will help sustain Pakistan’s economy. A study (Zia et al., 2018) claimed that CPEC-related project is not only providing employment opportunities but also helps in enhancing the capacity of domestic human resources as well.

The literature supported the relationship between HCI and EG (Bryant and Javalgi, 2016). Glaeser et al. (2004) found that HC is more effective than institutions in generating EG. O’Mahony (2012) found that continuous learning and formal education contributed toward building HC that, in turn, contributed to EG. Olimpia (2013) developed a way to compute the value of HCI in OECD countries and resulted that HCI contributed toward EG despite the different levels of competencies and efficiencies among different countries. CPEC is not only focused on transport infrastructure, telecommunication, and energy infrastructure but also focuses on the necessary physical infrastructure of the whole region (Iqbal et al., 2019a). Out of 46 billion approximately 13.58 billion USD was expected to be spent on infrastructure. As per (Zia et al., 2018) almost 52, 000 direct jobs were created under 6 CPEC-related road infrastructure projects. They also negated the myth of the Chinese getting more employment opportunities in CPEC projects. It is argued by authors (Xia et al., 2022) that FDI and gross capital formation lead to human capital development in both the short and long run, subsequently leading to the economic progression of the country. Jakhon (2021) claimed that there is a close association between the workforce and the economic progress of a country. Abid et al. (2020) claimed that organizations should work on their employee’s skillsets to achieve sustainability. It is also stated (Hosan et al., 2022) that any country can benefit from investments in IT or the economy if its employees are more skilled. A decrease in the skilled, motivated, and knowledgeable workforce will result in a slowdown in economic activities (Naeruz et al., 2022).

Roads and transportation infrastructure plays an important role in the economic growth of a country Zhang and Levinson (2007). The researchers (Ali et al., 2018a) used a questionnaire to determine the attitude of local people toward roads and transport infrastructure built under CPEC.

The results indicated a strong socio-economic impact of road infrastructure that in turn affected local people’s attitude toward CPEC projects. Pakistan ranks at 105th position in overall infrastructure in 2019 as compared to 93rd in 2018 and 100th in 2017, its ICT adoption has also faced a decline from 127 to 131st position from 2018 to 2019 Haider (2019, Oct 10). The decline in the overall quality of infrastructure has affected adversely its economic competitiveness. Despite serious concerns for the development of infrastructure, this sector was always neglected historically (Mehar, 2017). Under CPEC, 3,000 km of networks of roads, railway roads, fiber optics, and oil pipelines are expected to be built (Ali et al., 2018b). Under CPEC, a 100-km road is planned to be built between Karachi and Lahore and an approximately 2,700-km highway between Kashgar to Gwadar. Moreover, many highways will be upgraded to improve Pakistan’s connectivity with China and some neighboring countries (Ranjan, 2015). Gwadar port will be used as headquarter for China and a hub for Pakistan to facilitate its trading throughout the world (Mahmood et al., 2020).

This widespread network of infrastructure is going to help the local trader in exports by providing ease of connectivity and saving their costs (Abid and Ashfaq, 2015). Extensive literature is available on the contribution of infrastructure toward economic growth (Estache and Iimi, 2008; Sahoo and Dash, 2009). The USA observed an increased volume of bilateral trade by enhancing its port efficiency (Clark et al., 2004). Improved infrastructure attracts FDI and increases the volume of trade (Edmonds and Fujimura, 2008). The researchers (Jebran et al., 2018) claimed that terms of trade have a positive impact on Pakistan’s economy using the ARDL regression model. The reason behind China’s prosperity and growth in the last decades is largely attributed to the huge development of its physical infrastructure (Straub, 2008). This supports the argument that the development of infrastructure in Pakistan under CPEC is going to open a new horizon of prosperity, growth, and fortune for its people (Qureshi, 2015).

In this modern era of technology, no government is ignorant of the fact of how ICTs can play a prime role in the dissemination of information that will subsequently create awareness regarding socio-political and ecological issues (Ali, 2018). The use of ICTs has witnessed a steady increase globally, from 400 million users in 2003 to 3.2 billion users in 2015 (ITU, 2015; Iqbal et al., 2019b).

A lot of research has focused on the contribution of IT investment to the economic growth of the countries. The authors (Dedrick et al., 2003) investigated the economic performance of a country in terms of profitability, labor welfare, and economic growth in response to the investment in the information technology sector. Some researchers (Piatkowski, 2004, 2006; Jalava and Pohjola, 2008; Vu, 2011; Ahmed and Ridzuan, 2013) argued that economic growth can be stimulated through ICTs. Besides investing in roads and railways, CPEC is also aimed at strengthening information connectivity in Pakistan. Construction on the Pakistan-China fiber optic project has started in May 2016 to expand the telecommunication structure in Gilgit Baltistan, while focusing on facilitating Pakistan with the 5th route for telecommunication traffic (Economic Times, 2016).

## Methodology

This article attempts to estimate the impact of foreign direct investment, human capital, information and communication technology, and transport infrastructure on Pakistan’s economic growth, in connection with CPEC, by taking data from FY-1985 to FY-2018. CPEC regional integration analysis calls for including other countries in the analysis, but the approximation of the implicit impact of CPEC is limited to Pakistan’s economy only.

### Data

To analyze the relationship between exogenous variables and economic growth we attempt to approximate the determinants of economic growth. GDP per capita in US$ is used as an endogenous variable. The exogenous variables used for this purpose are foreign direct investment net inflow in US$ (FDI), human capital (HC), fixed telephone subscription (FTS), and density of roads (DRO). The annual time series data is used covering the period from 1985-to 2018. All relevant variables data is taken from World Development Indicators (WDI) and Penn World Table (PWT). After that, a natural logarithmic transformation was used to normalize the data for further estimation. Sensitivity analysis is done by incorporating other macroeconomic variables, such as central government debt, real interest rate, real effective exchange rate, and the unemployment rate in the model, but the results are robust even without the inclusion of these variables. The variable’s description and their specification for the empirical analysis are as follows given in Table 1.

**TABLE 1:** Definition of variables.

Variable	Proxy	Definition	Source
Economic Growth	GDP per capita growth (annual %)	natural log real GDP per capita in US$	Dhrifi, 2015
Foreign Direct Investment	Foreign Direct Investment (FDI)	Net inflow of foreign direct investment in US$	Dhrifi, 2015
Human Capital	Human Capital Index (HCI)	Human Capital Index from WDI measures the contribution of education and health toward the productivity of next generation of workers	Ederer et al., 2007
Transportation Infrastructure	Density of Roads (DRO)	Total network of roads divided by total land area of the country	Hong et al., 2011
Information Communication Technology	FTS	Sum of active telephone lines voice over-IP and wireless local loop and fixed payphone	Sridhar and Sridhar, 2007

### Data Analysis

The time-series data shows the trendy behavior, i.e., deterministic or stochastic. There may be a problem of non-stationarity in time series data and approximated parameters are spurious, and it is essential to eliminate this problem for best linear unbiased estimator (BLUE) parameters. In the first step, the unit root is checked and the stationarity of the variables at I (0), or I (1) is identified. There are enormous unit root tests in econometrics literature for time series data and this analysis checks the stationarity of data through Augmented Dickey and Fuller (1979) and Phillips and Perron (1988) unit root tests. The results of these tests will help to select the appropriate econometric regression specification.

#### Unit Root Test

The Augmented Dickey-Fuller test diagnosed the stationarity or non-stationarity of the variable. The general equation with time and trend applied for the ADF test is as follows:


(1) $$△\,Y_{i}=\beta_{0}+\beta_{1}\,t+\alpha \,Y_{t-1}+\sum_{t=1}^{m}\rho_{i}\,△\,Y_{i-1}+\varepsilon_{t}\,\ldots \,\ldots$$

Whereas △*Y*_*i*−1_ is lag difference term, β_0_ is a constant term and *t* is time trend. To incorporate lag difference in the model, no serial correlation was found among errors ε_*t*_ terms. The ADF test used additional lags of the first differenced variable. The Phillips and Perron (1988) test estimates the same equation used for checking the stationary, but they contain the first lag and difference in the unit root regression. The large negative values support accepting the null hypothesis, i.e., the series has a unit root, and the alternative is vice-versa. To check the stationarity variables, Augmented Dickey-Fuller (ADF) and Phillips-Perron (PP) unit root tests are used, and estimated results are presented in the following Table 2.

**TABLE 2:** Unit root test.

Variables	ADF fisher (chi-square)	PP fisher (chi-square)	Decision
**Intercepts with no trends at first difference**			
GDP	−5.2277 (0.0002)	−5.2277 (0.0002)	Stationary
FDI	−4.6056 (0.0009)	−4.6541 (0.0007)	Stationary
HCI	−5.2835 (0.0002)	−5.2901 (0.0000)	Stationary
FTS	−3.3021 (0.0232)	−3.2549 (0.0258)	Stationary
DRO	−2.7366 (0.0811)	−2.7447 (0.0798)	Stationary

*The reported results referred that all appropriate variables have unit root properties and stationery at integrated order I (1). The null hypothesis for both ADF fisher and PP fisher is rejected at first difference.*

#### Cointegration

A further step is to investigate common stochastic trends or cointegration among variables because growth is a long-running phenomenon rather than the short run. This research uses Johansen (1988) and Johansen and Juselius (1990), and Engle and Granger (1987) cointegration test. These tests indicate a long-run relationship in time series data. These tests estimate multiple heterogeneous cointegration vectors, i.e., preferred over traditional time series cointegration technique with the null hypothesis that “no cointegration” exists among variables.

#### Johansen Cointegration Test

As proposed by Johansen (1988) and Johansen and Juselius (1990), there are two test statistics for checking the cointegration vectors in the model. The Johansen test can be seen as a multivariate generalization of the Augmented Dickey-Fuller test. This generalization estimates the linear combinations of variables for unit roots. The estimation procedure of this test is to examine the cointegrating vectors through the Trace test and Eigenvalue. The trace test supports the *H_0_*, which implies the number of cointegrating vectors equal to (0, 1, 2, 3…). and the Eigen value checks the presence of cointegrated vectors against *H_A_*. Therefore, *H_0_* for testing numbers of cointegrated vectors can be set as *H*_0_=0 against the *H*_1_=1 and *H*_0_=1 against the *H*_*A*_=1, and so on (Khalafalla and Webb, 2001; Dritsakis, 2004). In other words, among five variables there are three variables with unit roots and, at most, two cointegrating vectors exist. This relationship is depicted as follows:


$$△\,Z_{t}=\emptyset +\Gamma_{1}\,△\,Z_{t-1}+\Gamma_{2}\,△\,Z_{t-2}\,\ldots \,\ldots \,\ldots +\Gamma_{p-1}$$

(2) $$△\,Z_{t-p+1}+\Pi \,Z_{t-1}+\mu_{t}\,\ldots \,\ldots \,\ldots \,\ldots \,\ldots \,\ldots$$

#### Engle and Granger Cointegration Test

The cointegration vector is unknown and another way to test the existence of cointegration is the residual-based static regression method, i.e., purposed by Engle and Granger (1987). The obtained residuals are tested for the presence of unit-roots. When the estimated residuals are stationary, cointegration exists among variables. In this case, an appropriate model for long run estimation is an error correction mechanism shown below:


(3) $$△\,{\hat{\mu }}_{t}=\rho \,{\hat{\mu }}_{t-1}+\sum_{j=1}^{k}\beta_{j}\,△\,{\hat{\mu }}_{t-j}+\varepsilon_{t}\,\ldots \,\ldots \,\ldots \,\ldots \,\ldots \,\ldots$$

The outcome of the unit root is very important to check before estimating the long-run equation.

Two prominent cointegration tests check the long-run relationship among variables or not at I (1), i.e., Johansen Cointegration and Engle-Granger.

In the Johansen Cointegration test, interpretation is made based on trace statistics and maximum eigenvalue statistics. These values verified the cointegrating vector among variables. In the case of this study, there exists an exclusive long-running association between gross domestic product, foreign direct investment, human capital, fixed telephone subscription, and density of road at “none as well as at most one”. The Johansen Cointegration test estimates multiple equations for checking the long-run relationship, but the Engle-Granger test estimates a single equation. The reported results in Table 3 confirmed that exogenous, as well endogenous variables, have a long-running relationship at a 5% significance level. In other words, gross domestic product, foreign direct investment, human capital, fixed telephone subscription, and density of road are cointegrated and have a long-running equilibrium. In the literature, multiple regression specifications exist for empirical estimation such as ARDL, VAR, and VECM. However, this analysis uses FMOLS in the empirical estimation because it can be used to estimate long-run coefficients.

**TABLE 3:** Johansen cointegration test.

Johansen cointegration test					

**Liner Deterministic Constant Trend**			**Number of Observation: 32**		

**Sample Size: 1987-2018**			**Lags (at first difference) 2**		

**Trace Test**					
**Maximum Rank**	**Parameters**	**Eigenvalue**	***t*-statistics**	**5% critical** **Value**	**Probability**
None	30	0.85804	100.1180*	69.81889	0.0000
At most 1	39	0.76883	51.31261*	47.85613	0.0228
At most 2	46	0.26671	14.69725	29.79707	0.7993
At most 3	51	0.15296	6.942031	15.49471	0.5844
At most 4	54	0.10566	2.791831	3.841466	0.0947

**Maximum Eigenvalue**					

**Maximum Rank**	**Parameters**	**Eigenvalue**	**Max-Eigen Statistics**	**5% critical** **Value**	**Probability**
None	30	0.85804	48.8053*	33.8768	0.0004
At most 1	39	0.76883	36.6153*	27.5843	0.0027
At most 2	46	0.26671	7.75522	21.1316	0.9181
At most 3	51	0.15296	4.15019	14.2646	0.8432
At most 4	54	0.10566	2.79183	3.84146	0.0947
**Engle-Granger Cointegration**					
	**Test Statistic**	**1% Critical Value**	**5% Critical Value**		**10% Critical Value**
**z(t)**	−5.245	−5.711	−4.881		−4.482

*All variables have a long-run relationship at a 10% significance level.

*Cointegration vector exists among variables.*

### Regression Specification

In the real world, every economy tries to grow at a faster rate than earlier, and scholars have been interested in the rate at which the economy is amplified. In this regard, investment in human capital, transport, telecommunication infrastructure, and FDI are being considered a determinant of economic growth for analysis as these are the major areas in which China is investing in Pakistan through CPEC.

The study adopts the Cobb-Douglas production function as follows:


(4) $$Y_{t}=\alpha_{0}\,X_{t}^{\beta }\,\mu_{t}\,\ldots \,\ldots \,\ldots \,\ldots \,\ldots$$

Whereas *X* is a vector of explanatory variables and β is a vector of parameters for period *t* = 1, 2,.,*T*. Taking the log of the above equation and transforming the function as follows we get:


(5) $$y_{t}=\alpha_{0}+\beta^{\prime }\,x_{t}+\mu_{t}\,\ldots \,\ldots \,\ldots \,\ldots \,\ldots$$

double log-linear regression specifications are as follows:

$$l\,n\,G\,D\,P_{t}=\alpha_{0}+\beta_{1}\,l\,n\,F\,D\,I_{t}+\beta_{2}\,l\,n\,H\,C_{t}+\beta_{3}\,l\,n\,F\,T\,S_{t}$$

(6) $$+\beta_{4}\,l\,n\,D\,R\,O_{t}+\mu_{t}\,\ldots \,\ldots \,\ldots \,\ldots \,\ldots \,\ldots \,\ldots \,\ldots \,\ldots$$

Whereas *lnGDP* is the natural log real GDP per capita in US$ (Hong et al., 2011; Dhrifi, 2015) and *lnFDI* is a net inflow of foreign direct investment in US$ (Mun et al., 2008; Dhrifi, 2015). The variable *HC* is the human capital index to have smoothly stimulated economic growth (Ederer et al., 2007); whereas *lnFTS* fixed telephone subscriptions as a proxy of information and communication technology in the natural log (Sridhar and Sridhar, 2007) and *lnDRO* density of roads used as a proxy of transport infrastructure in the natural log (Hong et al., 2011).

#### Fully Modified Ordinary Least Squared Regression

In the empirical estimation, the presence of cointegration is usually measured through using two regression technique that has been based on the OLS method, i.e., fully modified ordinary least squared (FMOLS) or dynamic ordinary least squared (DOLS). These regression specifications, FMOLS and DOLS, were developed by Phillips and Hansen (1990) and Stock and Watson (1993), respectively. FMOLS adopts the semi-parametric method for the approximation of long-run parameters. This technique gives asymptotically efficient and reliable coefficients even when the sample size is small. Similarly, this technique solves the problem of endogeneity, serial correlation, and heterogeneity in the long-run parameters (Agbola, 2013; Al-Mulali et al., 2014; Bashier and Siam, 2014; Fereidouni and Al-Mulali, 2014). FMOLS estimates a single cointegration equation for all exogenous variables cointegrated with a time trend. Amarawickrama and Hunt (2008) reported that the FMOLS technique makes an appropriate correction to the inference problem in Engel and Granger’s cointegration methodology and, hence, the long-run approximated parameters are valid. The FMOLS allows consistent and efficient estimator in the presence of cointegration, and, at the same time, it indicates the problem of nonstationary, as well as simultaneity biases in the heterogeneous cointegrated variables. The estimated parameters from FMOLS are unbiased due to endogeneity determined in the I (1) and the model can be written as follows:


(7) $$y_{t}^{+}=y_{t}-{\hat{\omega }}_{12}\,{\hat{\Omega }}_{22}^{-1}\,{\hat{\mu }}_{2\,t}\,\ldots \,\ldots \,\ldots \,\ldots \,\ldots \,\ldots \,\ldots \,\ldots \,\ldots .$$

where ${\hat{\mu }}_{1\,t}$ is the residual of the cointegration equation estimated by OLS and ${\hat{\mu }}_{2\,t}$ are the differenced residual regressors equations or the residual of the differenced regressors equations. The FMOLS estimators and their covariance are given by:


(8) $$\hat{\theta }=\left[\begin{array}{c} \hat{\beta }\\ {\hat{\gamma }}_{1} \end{array}\right]=\left[\sum_{t=1}^{T}z_{t}\,z_{t}^{\prime }\right]\,\sum_{t=1}^{T}z_{t}\,y_{t}^{+}-T\,\left(\begin{array}{c} {\hat{\lambda }}_{12}^{{+}^{\prime }}\\ 0 \end{array}\right)\,\ldots \,\ldots$$

(9) $$v\,a\,r\,\left(\hat{\theta }\right)={\hat{\omega }}_{12}\,\left[\sum_{t=1}^{T}z_{t}\,z_{t}^{\prime }\right],{\hat{\omega }}_{12}={\hat{\omega }}_{11}-{\hat{\omega }}_{12}\,{\hat{\Omega }}_{22}^{-1}\,{\hat{\omega }}_{21}\,\ldots \,\ldots$$

where ${\hat{\lambda }}_{12}^{+}={\hat{\lambda }}_{12}-{\hat{\omega }}_{12}\,{\hat{\Omega }}_{22}^{-1}\,{\hat{\Lambda }}_{21}$ are called bias correction terms and $z_{t}={\left(x_{t}^{\prime },d_{1\,t}^{\prime }\right)}^{\prime }\,{\hat{\omega }}_{12}$ is the estimation of long-run covariance of μ_1*t*_ conditional on μ_2*t*_.

For analysis FMOLS developed by Phillips and Hansen (1990) is used. The advantage of this technique allows for greater flexibility in the presence of heterogeneity in the cointegration vectors. Another advantage lies with the interpretation of the point estimates if cointegrated vectors are not homogenous. This study uses FMOLS as it allows consistent and efficient estimator in the presence of cointegration (Olofin et al., 2019; Peng and Wu, 2020; Srivastava and Talwar, 2020), and, at the same time, it indicates the problem of nonstationary and simultaneity biases in the heterogeneous cointegrated variables.

The following Table 4 shows the long-run parameters from FMOLS. The overall performance of the regression specification is seemingly good because 80% of the variation can be explained and most of the coefficient signs are consistent with the theory, as well as prior empirical studies. Consequently, the *t*-stat of the explanatory variables shows that all variables are relevant to the model, except the independent variable of the density of road, which has often been found insignificant in some of the studies. The variables are transformed into natural logarithm for normalizing them. The expected sign of FDI is related to prior literature and estimated parameters have low elasticity due to low coefficient value, where one percentage change in FDI can bring a change in gross domestic product up to sixteen percent.

**TABLE 4:** Fully modified least squares (FMOLS).

Variable	Coefficient	Std. Error	t-Statistic	Prob.
FDI	0.165849*	0.089518	1.852690	0.0753
HCI	0.2780112***	0.804581	3.455354	0.0019
FTS	−0.482846**	0.239520	−2.015889	0.0543
DRO	0.1041746	1.028182	1.013192	0.3203
INTERCEPT	5.523363	2.764101	1.998250	0.0562
R-squared	0.829026	Mean dependent variance		6.480247
Adjusted R-squared	0.802722	S.D. dependent variance		0.473103
S.E. of regression	0.210133	Sum squared residuals		1.148055
Long-run variance	0.068544			

*, **, and *** show that the coefficients are significant at 10 percent, 5 percent, and 1 percent level of significance Method: Fully Modified Least Squares (FMOLS) Cointegrating equation deterministic: C Long-run covariance estimate (Bartlett kernel, Newey-West fixed bandwidth = 4).

The second predictor variable is very important in the point of explicit economic growth, i.e., investment in human capital. The sign of the human capital coefficient also supports the prior literature as well as the theory. The calculated coefficient has a large value and positive relationship with GDP; whereas one percentage change in HCI can raise the gross domestic product by twenty-seven percent and the slope coefficient is also highly significant. Correspondingly, enormous literature is available on estimating the impact of human capital on economic growth with the help of other proxy variables, such as Gross or Net enrollment ratio, but complete data on the human capital index is easily available, and HCI is used in this analysis (Ederer et al., 2007).

There are numerous proxies available for the approximation of the relationship between economic growth and technology. Prior literature has used different proxies, such as household internet connection, mobile cellular subscription, and individual internet usage (Iqbal et al., 2019b; Asongu and Odhiambo, 2020). Most of the studies empirically estimate technology as a control variable because they indicated that this indicator has an implicit impact on economic growth. At present, there is no ambiguity that the role of technology rapidly boosts economic growth and this study used fixed telephone subscriptions as a proxy for technology. The estimated coefficient sign is negative and not inimical for economic growth, whereas percentage change in fixed telephone subscriptions significantly deteriorates gross domestic product by forty-eight percent and the slope coefficient is statistically highly significant. The proxy of transport infrastructure is the density of road and researchers used different proxies for this, such as the length of the road, railway carriages, etc. In the prior literature, the researcher verified that role of transport infrastructure plays a vital role in sustainable economic development. So, in other words, transport help to boost implicit economic growth, and the estimated coefficient is positively affiliated in the case of this research. The estimated value seems good but insignificant (Diaz et al., 2016). Economic growth has a multidimensional aspect and a lot of fundamental variables possible in the empirical estimation, but the estimated slope coefficient gives an appropriate outcome due to data constraints in this study being limited on these variables.

The residual diagnostic tests, such as LM, Q-Statistics, Normality Test, Breusch-Pagan-Godfrey, and White Heteroscedasticity tests, describe the intensity of regression specification. The calculated statistics of these tests are favorable for the estimated regression specification. Therefore, the causality among variables is also checked and found that most of the variables have a unidirectional relationship with each other. In the sensitivity analysis, the long-run parameters were also approximated with the help of an error correction mechanism and no significant change was observed, the reason why estimated coefficients from FMOLS are more reliable.

## Discussion

The key objective of this research is to empirically approximate determinants of the economic growth of Pakistan from 1985 to 2018. Enormous literature is available on determining economic growth using different exogenous variables with different proxies, but this research attempts to integrate all major variables in which CPEC is going to invest. It is aimed at estimating whether the sectors in which CPEC is going to invest will empirically help in boosting Pakistan’s economy.

Several empirical investigations and theoretical studies have been conducted on FDI and EG (Dhrifi, 2015). The positive impact of FDI on EG is widely recognized in the theoretical literature, but mixed results are prevalent in the empirical investigation over the past 20 years using a simultaneous equation model. Similarly, Hansen and Rand (2006) argued that, theoretically, FDI plays a substantial role in the growth of developing countries. However, literature regarding the negative impact of FDI is also available. Falki (2009) argued that the negative impact of FDI can redress the positive effects of FDI. Another researcher (Lipsey, 2004) argued that the positive effects of FDI are more in just country. Research shows mixed results for each variable’s contribution to economic growth. A negative relationship between FDI and economic growth (Hye, 2011; Ray, 2012; Akalpler and Adil, 2017) and a positive nexus between FDI and economic growth (Chaudhry et al., 2013; Kalai and Zghidi, 2019). The results of this study are in line with previous studies (Azman-Saini et al., 2010; Chaudhry et al., 2013; Dhrifi, 2015; Kalai and Zghidi, 2019), showing a positive significant impact of FDI on the economic growth of Pakistan. This result also conforms to the study by Baiashvili and Gattini (2020), claiming an increased effect of FDI on low- to middle-income level countries than on high-income countries.

The result showed a significantly positive impact of human capital on the economic growth of Pakistan. The estimated coefficient of human capital contributes to economic growth by 27%. This is also in accordance with previous research (Bryant and Javalgi, 2016; Maitra, 2016; Musibau et al., 2019; Amna Intisar et al., 2020; Matousek and Tzeremes, 2021). The human capital of any country is one of the strategic factors for enhancing its economic growth. It is claimed (Affandi et al., 2019) that medium- to long-run economic growth can be achieved by investing in human capital as human capital can help in building cognitive skills, which, in turn, enhances the quality of the labor force participating in developing economy in various regions. Among all other variables, human capital seems to contribute more to the economic growth of Pakistan. The benefits of all investments coming through CPEC can be realized if Pakistan develops and trains its labor with the required knowledge and skills. Thus, it can be said that human capital directly affects economic growth by expanding knowledge and skills.

The infrastructure also plays a vital role in the economic development of a country. To approximate the impact of transport and telecommunication infrastructure, this study uses a proxy of the density of roads and ICT, respectively. The appropriate proxy of ICT used in empirical estimation is a fixed telephone subscription. In this era of globalization, ICT is considered one of the prime factors that can contribute to the economic development of countries. However, few studies support this theory. A recent study (Soomro et al., 2022) has shown a positive impact of ICT on economic growth in few countries, whereas negative in others. Previous studies have shown a bidirectional relationship between telecommunication infrastructure and economic growth in high-income countries (Shiu and Lam, 2008; Farhadi et al., 2012), and a unidirectional relationship between these two was found (Datta and Agarwal, 2004; Yousefi, 2011). However, as Pakistan is one of the developing countries that is still striving to enhance its information and communication technology infrastructure, the results of the analysis are in line with the previous literature (Yousefi, 2011), showing negative relation between ICT and the economic growth of Pakistan.

Transportation infrastructure helps in boosting economic activity. There is a time gap between which transportation infrastructure is being built and benefits start to be realized. Therefore, the literature regarding the mixed results of transportation infrastructure on economic growth is also available; an insignificant relationship between transport infrastructure and economic growth (Baum-Snow, 2013), whereas research on the positive impact of transportation infrastructure on economic growth is also available (Zhang et al., 2012; Chatman, 2014; Irshad and Ghafoor, 2022). It is also evident from the literature that transportation infrastructure contributes positively to the economy, but not all modes of transportation contribute equally to the economy (Hong et al., 2011; Diaz et al., 2016). The study shows an insignificant relationship between the density of roads and economic growth during the selected time frame. Although this study is in line with previous studies (Diaz et al., 2016), Pakistan needs to consider the route very carefully if it wants this project to provide the expected benefits.

## Conclusion and Implications

It is a widely held view that economic corridors bring several benefits to the region where they are being built. Whether be an increased economic growth or an enhanced living standard, the prosperity of a country or region is related to economic corridors. There is extensive literature available suggesting that new corridors being built in African countries have the potential to change the economic position of these countries. Similarly, as Asia is becoming a trade hub, the development of the economic corridor is necessary to fulfill the increased demands of trade (Hussain, 2017). The CPEC, a sub-project of OBOR, is also serving the same purpose. This research primarily focuses on identifying the factors where China is investing to investigate whether this investment will bring the desired benefits. The results glorify the importance of developing human capital to reap the benefits of investment coming under CPEC.

The research concludes that among the factors chosen human capital investment contributes the most to the development of the economy of Pakistan. The researchers (Musibau et al., 2019) claim that human capital development may lead countries toward sustainable economic growth. In countries with poorly developed education and capital markets, many qualified citizens may also be unable to find the proper skills to commit to their full potential for economic growth. The development of human capital assures foreign investment that will subsequently help in reducing poverty in developing countries and is prioritized by many researchers. CPEC is going to revolutionize the business sector of Pakistan as bilateral trade between Pakistan and China will increase. As indicated by our results, if Pakistan’s local business community wants to reap the benefits of CPEC, they must work on developing the human capital. It is claimed (Abid et al., 2020) that organizations can achieve sustainability by focusing on factors affecting long-term growth and by developing skillset and knowledge base of their human capital to deal with and adapting changes. These skillset enhances their make them optimistic which in turn increases their capabilities to thrive at work leading toward good human capital (Abid et al., 2021) and hence, economic growth of the country. One important aspect of the skillset of human capital, i.e., leadership, can also work wonders to utilize the benefits of CPEC to its fullest potential because ethical leadership builds the trust of employees resulting in improved work engagement and better productivity at workplace (Ilyas et al., 2020). Given the importance of human capital in reaping the benefits of CPEC investment, it is recommended to pay attention to developing a knowledge base, skillset, and fair perception of employees as it can result in subjective well-being of people (Abid et al., 2019, 2020).

One of the prime areas of investment is IT infrastructure. The role of IT investment in the economic growth of the countries cannot be denied. Although these results of the analysis show a negative relationship between ICT and Economic growth, which is in line with Farhadi et al. (2012) who states that countries with low-income levels have a weaker relationship between ICT and economic growth. One of the reasons for this relationship would be the use of the proxy as data is not available on the latest proxies.

A positive nexus between FDI and the economic growth of Pakistan during the chosen period is found, as FDI is attracted more to open economies with fewer rules and regulations and having a skilled workforce and opportunities to grow. The inflow of FDI is beneficial for the host country in terms of creating employment opportunities, creating a competitive market, raising exports, and advances in technology. This is going to be a positive contributor to the economy of Pakistan as CPEC is expected to bring massive investments to Pakistan and Pakistan would be ready to benefit from its spillover effects in many more years to come.

Quite contrary to many studies focusing more on the development of transport infrastructure for better economic growth, these results show an insignificant relationship between transport infrastructure and economic growth. This is largely due to the spatial effect of such investments and time lags between when these investments were made and when one country starts realizing the benefits associated with the investment. These results are also in line with Berechman et al. (2006) who claimed that without consideration of the time lag between when transport investments were made and the realization of benefits, the results obtained can have an element of bias in it. Therefore, Pakistan must wait for some time in order to gauge the empirical effects of transportation infrastructure on economic growth.

As CPEC is expected to enhance imports and exports of the country and specific products of regions, particularly in route of CPEC, private investors would benefit from the study in identifying those sectors whose imports and exports would benefit them the most and have an impact on the overall economy. This research will be useful for researchers to conduct a similar type of research at corridors level projects all around the world like OBOR. Human capital investment can transform a developing country into a productive population, therefore, policy makers are required to emphasize investment in education.

## Limitations and Future Recommendations

One of the limitations is the limited time span for study. This study can be replicated with an increased time span. Future studies can use these proxies with more reliable and complete data for selected variables for the increased time span. This research can be replicated with other proxies, or the use of indexes already developed. The study can include more variables like gross capital formation, trade openness, and exchange rates. It is yet to decide whether CPEC is going to have an implicit effect or an explicit one. To get a clear answer, the research can be done by increasing the period to gauge real times effects of CPEC-related investment. The recent economic scenario of the world has changed owing to the COVID-19 pandemic. Future studies may attempt to empirically investigate the impact of COVID-19 on CPEC investments and economic growth. The research can be extended to include the sustainability of the workforce, environment, and economic progression in the wake of CPEC before, during, and after COVID-19.

## Data Availability Statement

The original contributions presented in the study are included in the article/supplementary material, further inquiries can be directed to the corresponding author/s.

## Author Contributions

MF conceived the idea, collected the data, and prepared writeup of the study. Z-u-R performed analysis and written the methodology part. MS refined the writeup of the study and re-checked flow of the study. All authors contributed significantly for this study.
